# Technological and Nutritional Characteristics of Gluten‐Free Pasta Enriched With Tomato and Linseed By‐Products

**DOI:** 10.1002/fsn3.71105

**Published:** 2025-10-18

**Authors:** Gabriella Pasini, Carlos Gabriel Arp, Sabrina de Leo, Andrea Brandolini, Lorenzo Estivi, Alyssa Hidalgo

**Affiliations:** ^1^ Department of Agronomy, Food, Natural Resources, Animals and Environment University of Padova Legnaro Italy; ^2^ Centro de Investigación y Desarrollo en Ciencia y Tecnología de Alimentos (CIDCA) Facultad de Ciencias Exactas‐Universidad Nacional de La Plata, Comisión de Investigaciones Científicas de la Provincia de Buenos Aires, Consejo Nacional de Investigaciones Científicas y Técnicas Buenos Aires Argentina; ^3^ Consiglio per la Ricerca in Agricoltura e L'Analisi Dell'Economia Agraria‐Unità di Ricerca per la Zootecnia e L'Acquacoltura (CREA‐ZA) Lodi Italy; ^4^ Department of Food, Environmental and Nutritional Sciences (DeFENS) Università Degli Studi di Milano Milan Italy

**Keywords:** faba flour, glycemic index, rice flour, texture, total dietary fiber

## Abstract

In recent years, the market's demand for gluten‐free products has been steadily rising, and similarly, the interest in the functionalization of the food matrix to obtain healthier products has been increasing. Furthermore, there is a growing interest in valorizing agri‐food by‐products that still contain ingredients that enhance the nutritional properties of food. The objective of this study was to evaluate the cooking and texture quality parameters, starch digestibility, and total dietary fiber of gluten‐free pasta enriched with varying additions (10% and 15%) of two food industry by‐products: tomato waste powder and linseed cake powder. The results indicate that the enriched gluten‐free pasta samples exhibited improved nutritional profiles, although some quality parameters were at times affected. The linseed cake powder improved resistant starch content, lowered cooking loss, and increased adhesiveness more effectively than the tomato waste powder. Conversely, tomato waste powder‐enriched gluten‐free pasta had higher dietary fiber (6.5% and 8.7% with 10% and 15% addition, respectively) and increased cooking loss (by 100% and 131% for 10% and 15%, respectively), as well as lowered firmness (by 9% and 34% for 10% and 15%, respectively). Nevertheless, all enriched samples showed a significant reduction in their predicted glycemic index, which corresponded to the level of by‐product addition. This study demonstrates the potential of utilizing food industry by‐products to enhance the nutritional value of gluten‐free pasta, thereby supporting a circular economy in food production.

## Graphical abstract

Gluten‐free pasta enriched with tomato and linseed by‐products shows improved nutritional quality and reaches the conditions to be commercialized as “source of fibre” and “high fibre” products. The technological quality of such products meets the standards for the gluten‐free pasta industry.

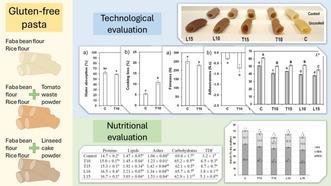

## 1 Introduction

The traditional pasta prepared with durum and/or soft wheat is not suitable for people with celiac disease, an inflammatory disorder characterized by injury to the lining of the small intestine after exposure to the gluten in durum and bread wheats, barley and rye in genetically predisposed individuals (Vaccino et al. 2009), and for other wheat‐related disorders such as wheat allergies and non‐celiac gluten sensitivity. Currently, the most effective therapeutic treatment for individuals affected by wheat‐related disorders is avoiding wheat and gluten products, and in the case of celiac disease, adhering to a life‐long gluten‐free (GF) diet (Størdal and Kurppa 2025).

Durum wheat pasta and bread wheat noodles are enjoyed worldwide for their rich flavor, ease of preparation, affordability, and long shelf life. However, wheat‐based pasta is not suitable for people with celiac disease, and the food industry is currently making pasta products from GF flours. Recent sharp increases in GF pasta demand have led to a global market worth about USD 1.52 billion in 2022 and, in the near future, the industry is expected to grow at a compound average growth rate ranging from 7.6% to 9.2% according to different market forecasts (Singla et al. 2024; Zion Market Research 2023). This sustained increase is further fueled by the fact that GF pasta is also sought‐after by non‐celiac people who exclude gluten‐based products from their diet due to wheat allergy and sensitivity issues, and even due to perceived health reasons (Singla et al. 2024; Winham et al. 2022).

Regrettably, GF products usually foster an excessive intake of carbohydrates and fats that, coupled with deficiencies in calcium, iron, fiber, vitamins B12 and D, zinc and magnesium, increase the risk of chronic ailments such as diabetes, obesity, and cardiovascular disease (Difonzo et al. 2022). The functionalization of GF pasta is considered a good strategy to improve the nutritional profile and prevent disease (Oladeji et al. 2025), similarly to what was recently done to traditional pasta through the inclusion of plant and animal material (Dziki 2021). Several authors have demonstrated that the addition of selected ingredients leads to an improvement in the nutritional characteristics of GF pasta. For example, Bento et al. (2021) determined that pre‐gelatinized carioca bean flour increased the protein and fiber content of GF pasta while maintaining good technological quality and sensory acceptance by consumers. Germinated chickpea flour was used by Sofi et al. (2020) to increase crude protein content, crude fiber, antioxidant activity, and total phenolic acids of GF noodles, while also reducing the glycemic index. Baah et al. (2022) produced GF pasta from maize and orange‐fleshed sweet potato and determined that the product had higher antioxidant properties but still maintained the desired quality parameters. Oniszczuk et al. (2019) increased the antioxidant activity of GF pasta with the addition of chestnut flour, detecting as many as 13 phenolic acids. Betrouche et al. (2022) used tomato and linseed waste to improve the tocols and polyphenols contents, as well as the antioxidant capacity, of raw GF pasta, and the gains were maintained in the cooked products (Estivi et al. 2024).

The manufacturing of functional pasta is also an efficient strategy to exploit natural and low‐cost resources, such as agri‐food by‐products, to design value‐added and innovative food products. The United Nations estimates that 1.05 billion tons of food are wasted globally, with more than 60% wasted at the household level (United Nations Department of Economic and Social Affairs 2025; United Nations Environment Programme 2021). In the European Union, over 59 million tons of food, representing around 132 kg of food waste per inhabitant, were wasted in 2022 (European Commission 2024). However, these neglected by‐products are still rich in nutrients and valuable natural bio‐compounds that can be recovered and exploited as functional ingredients by the pharmaceutical, cosmetic, and food industries (Gullón et al. 2020).

Tomato pomace, a by‐product of tomato processing that represents 2%–10% of the fresh tomato (Casa et al. 2021), is a good source of fiber (60–70 g/100 g dry basis), antioxidants (5–10 g/100 g dry basis), and protein (10–20 g/100 g dry basis) (Betrouche et al. 2022; David‐Birman et al. 2018; Elbadrawy and Sello 2016). Similarly, linseed cake, a by‐product of linseed oil extraction, has abundant protein and water‐soluble fiber (Betrouche et al. 2022; Mueller et al. 2010). Different authors have studied the enrichment of pasta with dietary fiber from agri‐food by‐products as an opportunity to increase consumer's fiber consumption. Simonato et al. (2019) determined that adding olive pomace to pasta significantly increased its dietary fiber content, influencing the rate of starch digestibility. Similarly, Tolve et al. (2020) found that enriching pasta with grape pomace decreased the rapidly digestible starch and increased the slowly digestible starch, causing a decrease in the in vitro predicted glycemic index. Regrettably, improving the nutritional features of these products often clashes with the goal of preserving sensory quality (Oladeji et al. 2025). While enriching GF pasta with different ingredients improves its nutritional value, it can also alter other characteristics, such as cooking loss, water absorption, firmness, and adhesiveness (Dziki 2021).

In previous studies, the antioxidant properties and antioxidant bioaccessibility of GF pasta enriched with tomato and linseed by‐products were evaluated, as well as some characteristics of the raw pasta, such as optimal cooking time, pasta fracturability, and partial nutritional composition (Betrouche et al. 2022; Estivi et al. 2024). However, information about quality parameters and other nutritional features (e.g., total dietary fiber content and starch digestibility) of the cooked pasta is still missing.

Therefore, the objective of this work was to determine how the enrichment of GF pasta with different quantities of two food industry by‐products, tomato waste and linseed meal, affects the cooking and textural parameters and the carbohydrate digestibility in final ready‐to‐eat products in relation to their composition.

## 2 Materials and Methods

### 2.1 Raw Materials

The tomato pomace powder (T) was produced by drying (45°C, 10 h) and grinding tomato pomace obtained from the Zimba Canning Company (Guelma, Algeria). The linseed cake powder (L), a residue of oil extraction by hydraulic press, was provided by Health Embassy Ltd (Cheltenham, England). The control GF pasta (C) was prepared from a blend of 66.7% rice flour (Bio Aglut Company, Constantine, Algeria) and 33.3% faba bean flour (Al‐Amir Company, Housh Essa, Egypt). The enriched pasta samples were manufactured by substituting 10% or 15% of the flour mix with tomato pomace powder (T10 and T15, respectively) or linseed cake powder (L10 and L15, respectively).

### 2.2 Pasta Making

**FIGURE 1:**
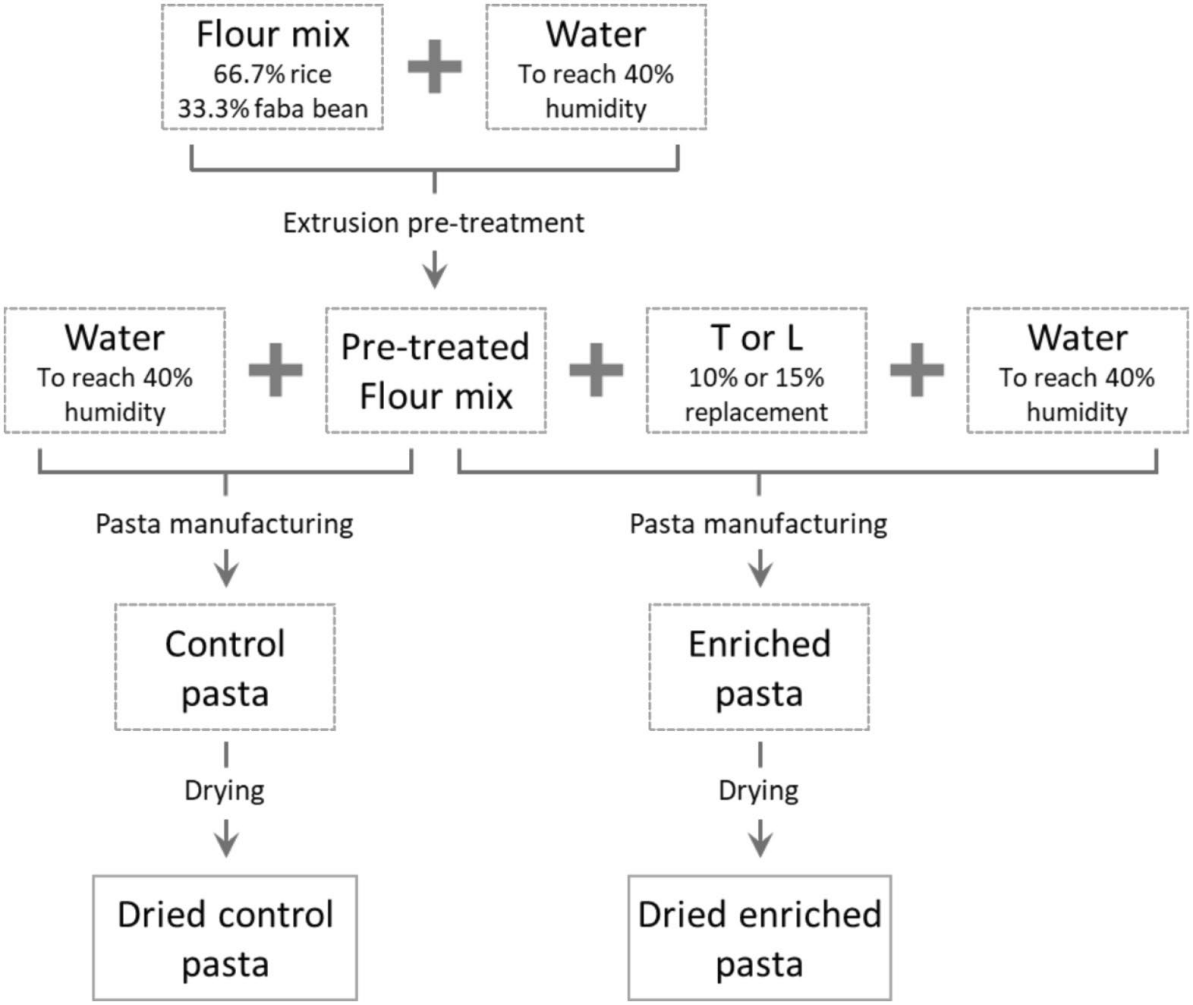
General flowchart of pasta samples production.

A flowchart of the pasta‐making procedure is depicted in Figure 1. Briefly, the control flour blend was mixed with water needed to reach 40% (*w*/*w*) humidity, and then pre‐treated with a PROGEL extruder (Braibanti, Milan, Italy) according to the following parameters: extrusion screw temperature 130°C; pellets outlet temperature 85°C–90°C; pellets extrusion pressure 10 bar; pellets length 5 mm. Short‐cut pasta (macaroni format) was immediately prepared from the pellets with a Mac30 pilot plant (Italpast, Parma, Italy) equipped with a pre‐kneading tank, a vacuum kneading tank, an extrusion cylinder, and a Teflon die.

The pre‐treated control flour mix alone, or mixed with the by‐product flours, was hydrated with water up to 40% humidity for 12–15 min and extruded under vacuum (−0.9 bar) at a pressure of 65–70 bar. The dough temperature was maintained at 40°C with a water‐cooled jacket. The pasta samples were dried in a Braibanti (Milan, Italy) experimental cell at low temperature (max. 60°C for 17 h), to limit heat damage and better preserve the nutritional characteristics of the final product (Giannetti et al. 2021). All the dry pasta samples were stored at room temperature until analysis.

### 2.3 Chemical Composition of Raw Materials and Pasta Samples

Moisture was determined according to AACC method 44‐15.02 (AACC International 2009f). Protein was quantified by the Kjeldahl method (AACC 46‐11.02, conversion factor 6.25) (AACC International 2009d). Lipids were determined by Soxhlet extraction using petroleum ether after acid hydrolysis of samples (AACC method 30‐10.01) (AACC International 2009c). Ashes were assessed according to AACC 08‐01.01 (AACC International 2009b). Total dietary fiber (TDF) content was measured as in AACC method 32‐05.01 (AACC International 2009g) with a dedicated kit (Megazyme, Wicklow, Ireland). Carbohydrates were computed by difference, and the total energy value (kcal/100 g) was determined using the following conversion factors: protein 4 kcal/g, carbohydrate 4 kcal/g, fat 9 kcal/g, and fiber 2 kcal/g. All chemical tests were performed in triplicate.

### 2.4 Determination of Pasta Quality Parameters

#### 2.4.1 Cooking Loss and Water Absorption

The optimal cooking time (CT), that is, the time needed to make the starchy white core of the pasta disappear, was previously determined as 8 min by Estivi et al. (2024) in a separate study. Cooking loss (CL, g solids/100 g of dry pasta) was evaluated according to the official method 66‐50 (AACC International 2009e) as the weight of the solids obtained by evaporating the pasta cooking water in a forced‐air oven at 110°C. Water absorption (WA) was measured as the percentage of weight increase after cooking the pasta. All determinations were carried out on three separate cooking batches.

#### 2.4.2 Color

The color analysis was carried out using a Chroma Meter CR‐II tristimulus colorimeter (Minolta Camera Co., Osaka, Japan) with a C standard illuminant according to the CIE‐L* a* b* system where the L* value represents the lightness ranging from 0 (black) to 100 (white), and a* and b* are chromaticity coordinates for red‐green and yellow‐blue components, respectively. The measurements were performed on four different samples of raw and cooked pasta. To assess color changes compared to the control (C), the following equation (Equation 1) was applied:

(1) $$∆E=\sqrt{{\left(L_{i}^{*}-L_{C}^{*}\right)}^{2}+{\left(a_{i}^{*}-a_{C}^{*}\right)}^{2}+{\left(b_{i}^{*}-b_{C}^{*}\right)}^{2}}$$

where *ΔE* is the total color difference, and the *i* and *C* subscripts for each L*, a*, b* parameter correspond to the *i*‐th sample and the control (C) sample, respectively.

#### 2.4.3 Texture

The textural properties of the cooked pasta were determined using a TA.XTPlus Texture Analyzer (Stable Micro Systems, Godalming, UK) supported with a 50‐kg load cell. A single piece of pasta (length of about 3.6 cm and height of about 1.0 cm) was placed on the lower plate and the rectangular probe (30 × 50 mm) was moved down onto the pasta surface (test speed: 2 mm s^−1^; deformation: 80%). Firmness was measured as the maximum force required to compress the cooked pasta sample, whereas adhesiveness, measured as a negative area, was the work required to withdraw the probe from the surface of the same sample. Data are the mean of ten measurements from three separate cooking replicates.

### 2.5 Starch Digestibility and Predicted Glycemic Index (pGI)

An in vitro procedure for starch digestibility was performed on minced cooked pasta samples using the AACC Method 32‐40.01 (AACC International 2009a), with minor modifications, as detailed by Romano et al. (2016). The hydrolysis rate for the starch digestion was expressed as the percentage of total starch (TS) as a function of time by spectrophotometrically measuring the amount of glucose using a D‐glucose assay kit (Megazyme, Wicklow, Ireland) after 0, 20, 30, 60, 90, 120, 150, and 180 min of incubation with pancreatic α‐amylase and amyloglucosidase. The rapidly digestible starch (RDS) and the slowly digestible starch (SDS) were calculated after 20 and 120 min of incubation. The data represent the mean of four different digestions. The TS content was calculated as the sum of the resistant starch (RS) and non‐resistant starch, quantified by a K‐RSTAR assay kit (Megazyme, Wicklow, Ireland). All the determinations were done in quadruplicate, and the results are expressed as g/100 g dry weight. The hydrolysis index (HI) was assessed by plotting the amount of glucose released from each sample vs. time, using white bread as a reference. The trapezoidal method was used to determine the area under the curve (AUC, 0–180 min) for each sample and for the reference. The HI was expressed as the ratio between the sample and the reference (Equation 2).

(2) $$\mathrm{HI}=\left(\mathrm{AUC}\;\text{test food}/\mathrm{AUC}\;\text{reference food}\right)\times 100$$

The predicted glycemic index was computed with the Goñi et al. (1997) formula (Equation 3).

(3) $$\mathrm{pGI}\;\left(\%\right)=39.71+0.549\times \mathrm{HI}$$

### 2.6 Statistical Analysis

The data were processed by one‐way analysis of variance (ANOVA) using the statistical software STATGRAPHICS Centurion (Statpoint Technologies Inc., Warrenton, VA, USA). Pearson's linear correlation analysis was performed with InfoStat (Di Rienzo et al. 2018).

## 3 Results and Discussion

### 3.1 Chemical Composition of Raw Materials and Pasta Samples

Table 1 shows the chemical composition of the raw materials used in the production of gluten‐free pasta. The nutritional value of rice flour relies mainly on carbohydrates, because the protein content was only 7.7 g/100 g, in agreement with the results described by Devraj et al. (2020) and Culetu et al. (2021). Faba bean flour had a protein content comparable to that of linseed meal, and both were within the range reported by De Angelis et al. (2021). Linseed also shows a good lipid content, comparable to that reported by Zarzycki et al. (2020), while tomato waste presented a high total dietary fiber content, as already observed by several authors (Betrouche et al. 2022; Del Valle et al. 2006; Teterycz and Sobota 2023; Zarzycki et al. 2020).

**TABLE 1:** Nutritional composition (g/100 g) of raw materials and raw gluten‐free pasta.

	Proteins	Lipids	Ashes	Carbohydrates^†^	TDF^‡^
Raw materials					
Rice flour	7.7 ± 0.3	0.68 ± 0.01	0.20 ± 0.01	79.4 ± 0.2	0.6 ± 0.1
Faba bean flour	28.2 ± 0.2	1.84 ± 0.01	2.87 ± 0.02	43.6 ± 1.8	11.9 ± 1.4
Tomato waste	16.1 ± 0.2	5.00 ± 0.16	4.04 ± 0.01	3.2 ± 1.8	62.9 ± 1.6
Linseed meal	27.0 ± 0.2	15.52 ± 0.01	5.09 ± 0.04	6.7 ± 2.5	37.5 ± 1.7
Gluten‐free pasta					
Control	14.7 ± 0.2^c^	1.47 ± 0.07^c^	1.06 ± 0.08^d^	69.0 ± 1.7^a^	3.2 ± 1.0^d^
T10	15.0 ± 0.1^bc^	1.45 ± 0.04^c^	1.23 ± 0.01^c^	65.2 ± 0.5^bc^	6.5 ± 0.3^b^
T15	15.3 ± 0.1^b^	1.92 ± 0.34^b^	1.42 ± 0.04^ab^	62.1 ± 0.5^d^	8.7 ± 0.7^a^
L10	16.5 ± 0.4^a^	2.21 ± 0.07^b^	1.34 ± 0.04^bc^	65.7 ± 0.7^b^	3.8 ± 0.1^cd^
L15	16.7 ± 0.1^a^	3.03 ± 0.04^a^	1.51 ± 0.04^a^	62.9 ± 1.1^cd^	5.1 ± 0.8^bc^

*Note:* All results are expressed as mean ± standard deviation, wet basis. For pasta samples, different letters in the same column indicate significant differences (*p* < 0.05).

^†^ Calculated by difference.

^‡^ Total dietary fiber.

**FIGURE 2:**
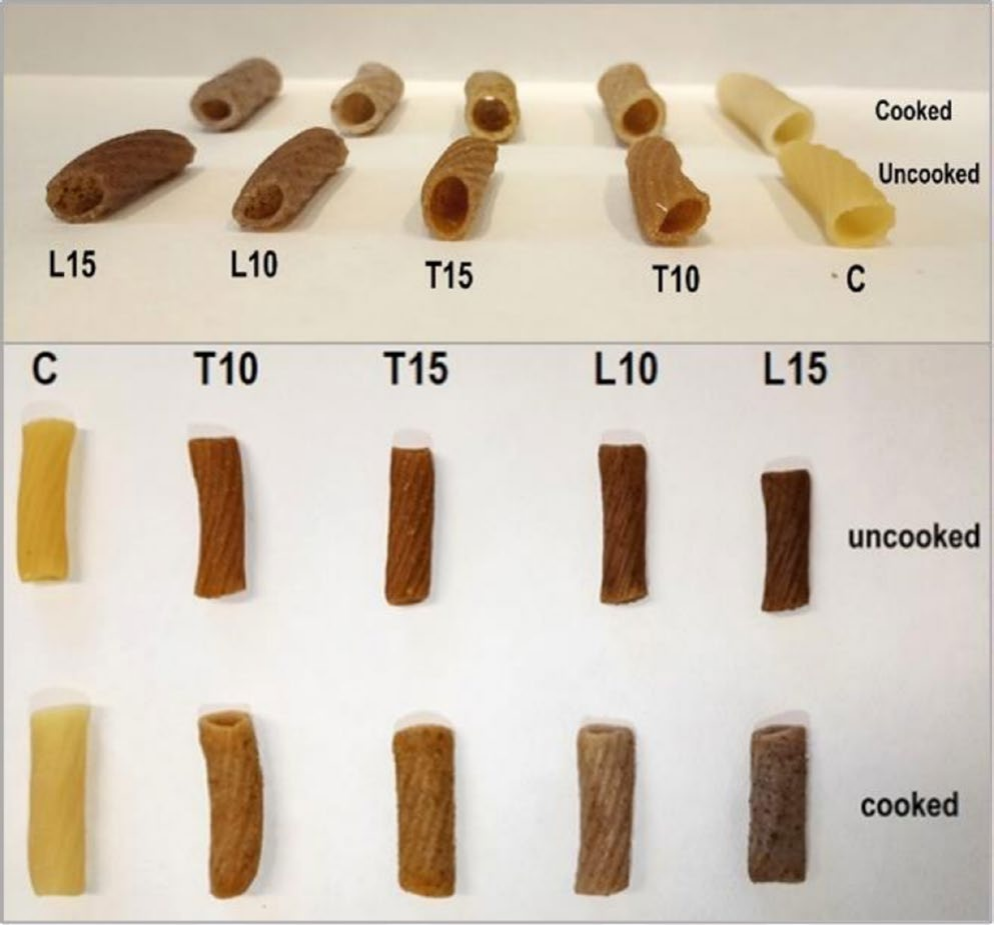
Cooked and uncooked samples of gluten‐free pasta.

The pasta samples obtained are shown in Figure 2, while their composition is reported in Table 1. All pasta samples presented similar moisture content (10.6%). The enrichment with the by‐products significantly increased the fiber content of all samples, and the highest value was reached with the addition of 15% tomato waste, an outcome similar to the durum wheat pasta enriched with the same percentage of tomato pomace (Padalino et al. 2017). The addition of 10% and 15% tomato waste powder to the pasta formulations achieved a dietary fiber content higher than 6.0 g/100 g of pasta, while the addition of the same levels of linseed cake powder led to dietary fiber values ranging between 3.0 and 6.0 g/100 g of pasta. Therefore, the T samples could be marketed with the claim “high fibre”, while the L samples should be considered as “source of fibre” according to the EC Regulation no. 1924/2006 (European Parliament and Council 2006).

Regarding the protein content, while the control and the T‐enriched pastas showed similar values, L enrichment provided significantly higher protein values, mainly due to the high protein content (27.0 g/100 g) of the linseed cake (Betrouche et al. 2022). This result is consistent with those found by Zarzycki et al. (2020) in semolina pastas enriched with 9%–17% linseed oil cake. Regarding the energy content of the samples, the pastas enriched with 10% and 15% T showed lower calorie content (346 ± 1 and 344 ± 1 kcal/100 g, wet basis, respectively) than the control (354 ± 2 kcal/100 g, wet basis) and the ones formulated with 10% and 15% L (356 ± 1 and 355 ± 1 kcal/100 g, wet basis, respectively). This agrees with the differences observed in the composition of the raw ingredients.

### 3.2 Determination of Pasta Quality Parameters

#### 3.2.1 Water Absorption and Cooking Loss

**FIGURE 3:**
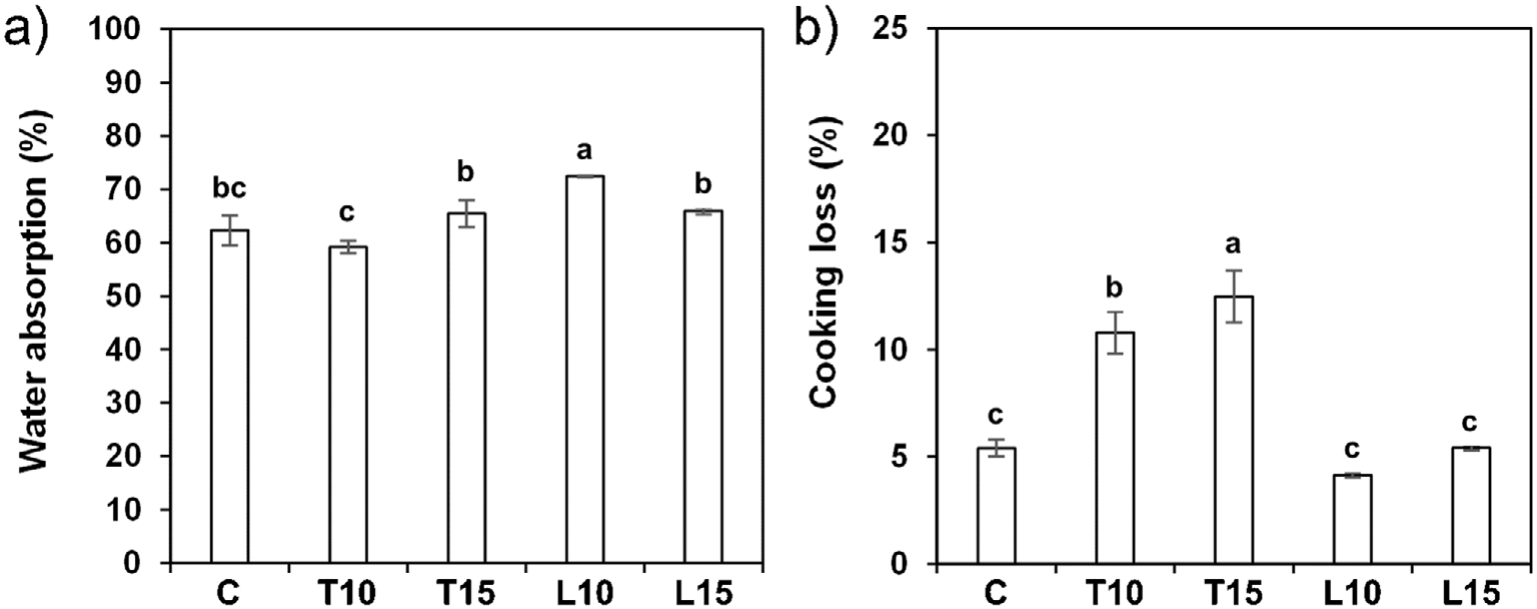
Quality parameters of gluten‐free pasta at the optimal cooking time (8 min): (a) water absorption and (b) cooking loss. Different letters indicate significant differences (*p* < 0.05).

Pasta's capacity to absorb water during cooking is affected by different parameters related to starch, fiber, protein, the swelling power of their constituents and their interaction. As shown in Figure 3a, although most enriched samples exhibited a slight increase in water absorption, only L10 pasta showed a statistically significant difference (*p* ≤ 0.05) compared to the control. This is likely due to its higher insoluble fiber content, whose hydroxyl groups facilitate water interaction through hydrogen bonding. These results are consistent with those from noodles with added fiber from wheat bran (Song et al. 2013). Moreover, the denaturation of proteins during heating can increase the accessibility for polar amino acid groups, improving the affinity for water, as demonstrated in legume proteins by Alonso et al. (2000).

For cooking loss, there are no significant differences between L10, L15 and the control pasta (Figure 3b). The protein and lipid quantities, higher in the linseed‐enriched pasta than in the tomato waste one, would favor coating and attaching the starch granules of the pasta matrix, preventing their leaching into the cooking water (Ye et al. 2018). However, Zarzycki et al. (2020) found that the replacement of semolina with flaxseed cake induced a slight decrease in cooking losses compared to the control sample; they attributed this effect to the presence in the flaxseeds of lignans and soluble fiber, which increase viscosity and reduce migration of dry matter components (e.g., starch) during cooking. Remarkably, the L10 and L15 samples had a cooking loss around 4%–5%, significantly inferior to the 8% value used as a threshold by the pasta industry for evaluating pasta acceptability (Desai et al. 2018). On the other hand, the enrichment with tomato waste induced an increase in the losses proportional to the percentage of replacement, probably due to the high content of insoluble fiber (Betrouche et al. 2022), which is known to weaken the starchy matrix in GF pasta from brown rice flour (Marti et al. 2010) and in wheat noodles enriched with insoluble fiber (Kim et al. 2017).

#### 3.2.2 Color

**FIGURE 4:**
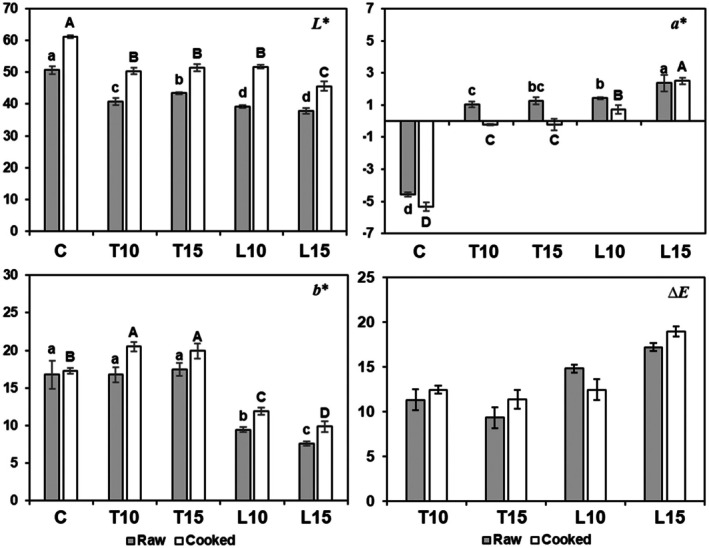
Color parameters L*, a*, and b*, and *∆E* for the raw and cooked pasta samples. Different lowercase letters indicate significant differences among raw samples, and uppercase letters indicate significant differences among cooked samples (*p* < 0.05).

Pasta color, a relevant element in consumer preferences, may be affected by several factors, such as ingredients and processing conditions. Statistically significant differences were observed between the control pasta and all the enriched samples because these samples showed L* lower than the control, exhibiting a darker appearance both raw and cooked. However, L* increased after cooking for all samples (Figure 4). On the other hand, a* shifted from a green tone (negative value in C pasta) to red (positive values in the other samples) due to the presence of natural pigments in the by‐products such as carotenoids and lycopene rather than chlorophylls. These results agree with those obtained by Teterycz and Sobota (2023) and Sinha and Manthey (2008). The positive values of b*, indicating yellow tones, were lower in L10 and L15 pasta in comparison to C, both raw and cooked. Control, T10, and T15 raw samples showed similar values. Nevertheless, both T10 and T15 displayed significantly higher values than the control after cooking (Figure 4). The color differences, as measured by the parameter *∆E* (Figure 4), were noticeable in all cases. As stated by Sanz et al. (2010) and Bellary et al. (2016), values of *∆E* higher than 3 can be spotted by consumers. In any case, consumer preferences for pasta color are variable and subject to familiarity and expectations (Lombardi et al. 2019). Many vegetables, fruits, and legume‐enriched pasta are already available on the market, which may contribute to an increase in familiarity and consumer acceptance of the different products. Nevertheless, pasta color can also be adjusted by using low quantities of additives without changing other technological parameters.

#### 3.2.3 Firmness and Adhesiveness

**FIGURE 5:**
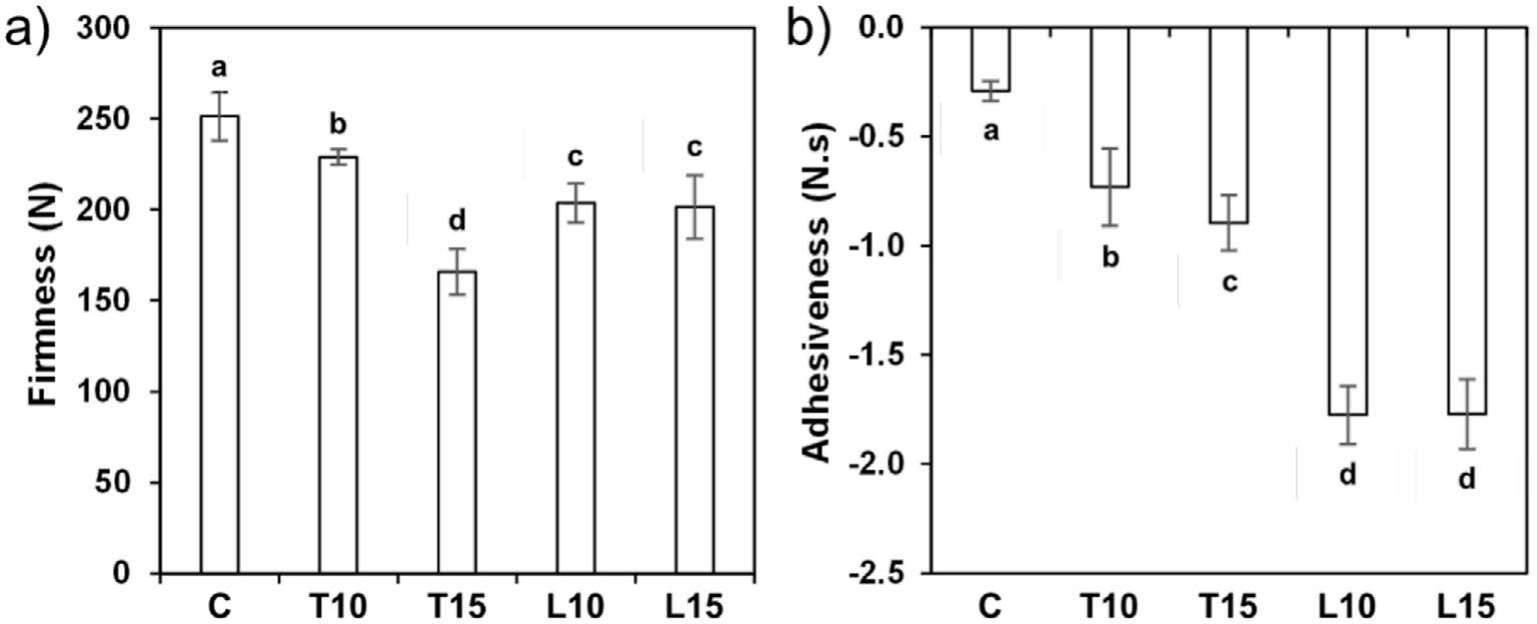
Textural parameters of cooked gluten‐free pasta: (a) firmness and (b) adhesiveness. Different letters indicate significant differences (*p* < 0.05).

All the pasta samples enriched with the by‐products saw a decrease in firmness compared to the control, as shown in Figure 5a. For the tomato waste‐enriched samples, firmness followed a trend contrary to fiber content, with T15 pasta (highest in fiber) showing the lowest firmness value. The inclusion of fiber in the starch matrix led to a weakened pasta structure, as also reported for rice‐based pasta by Marti et al. (2010). Linseed cake‐enriched pasta also showed lower firmness values than the control, although in this case the results were not affected by the enrichment level. These data agree with those reported for flaxseed cake‐enriched pasta by Zarzycki et al. (2020), for pasta with mango and apple peel flour (Jalgaonkar et al. 2018; Lončarić et al. 2014), and for vermicelli with apple, pear or date waste (Bchir et al. 2022). The addition of by‐products seems to undermine the structure and weaken the strength of the pasta. In fact, Bustos et al. (2011) observed that higher oat bran contents interfere with the development of a well‐formed protein‐starch matrix, probably due to the presence of the fiber, which tends to disrupt the gluten matrix organization, thus leading to lower firmness (Islas‐Rubio et al. 2014).

The adhesiveness of all the enriched samples was higher than that of the control sample (Figure 5b). In particular, the substitution with by‐products containing fiber (such as those from tomato) could weaken the starch structure of the pasta, thus more components would unbind and release during cooking, causing an increase in the adhesiveness (Kang et al. 2018). Similarly, the linseed by‐product had an even greater effect on pasta adhesiveness, which could be attributed to its high content of proteins, as a strong correlation was found between the adhesiveness and the protein content of the GF pasta samples (*p* = −0.988). Other authors have reported an increase in adhesiveness when enriching pasta with high‐protein ingredients (Aínsa et al. 2021). Additionally, some authors have reported a high content of soluble fiber in flaxseed cake powder (Zarzycki et al. 2020). Soluble fiber can retain water and generate viscosity, contributing to the adhesiveness of the products (Rakhesh et al. 2015).

### 3.3 Starch Fractions Determination and Predicted Glycemic Index

**FIGURE 6:**
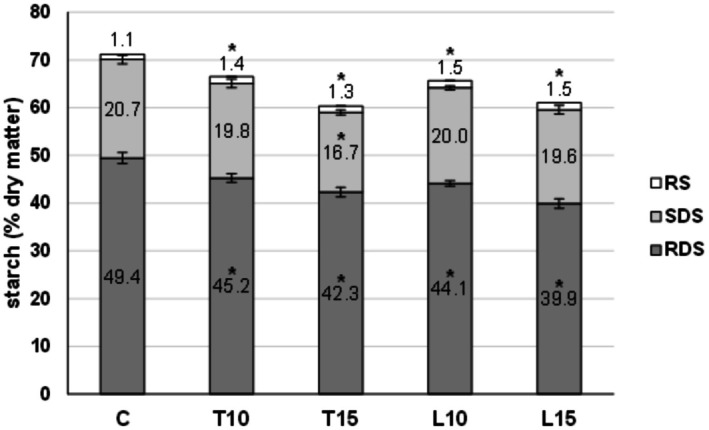
Total starch, resistant starch (RS), slowly digestible starch (SDS), and rapidly digestible starch (RDS) content of gluten‐free pasta. Numbers in the bars indicate the amount of each fraction. Values with * are significantly different from the control for the same fraction.

Figure 6 reports the percentages of starch fractions (RDS, SDS, and RS) of the control and of the four enriched pasta samples. A significant decrease in the rapidly digestible starch (RDS) was recorded, without difference between T and L enrichment, in accordance with the increase in by‐products content. In general, the lower glucose release could be due to the progressive starch reduction in pasta, as suggested by Rocchetti et al. (2020) and Simonato et al. (2019). In fact, although Aravind et al. (2012) and Gallo et al. (2020) state that starch losses during cooking can also contribute to decreasing the total starch or some of its fractions, in this case the starch proportions of the samples T10 and T15 (highest cooking losses) were similar to those of the samples L10 and L15 (cooking losses similar to the control), therefore suggesting that the dilution effect of replacing rice and faba flours with tomato waste and linseed cake powders is the main reason for these changes.

On the other hand, a significant increase of the RS fraction was evidenced in L and T pasta samples compared to the control, regardless of the replacement percentage. The linseed cake has a higher impact on the increase of resistant starch than the tomato by‐product (*p* < 0.05), probably due to its higher lipid, protein, and soluble fiber content. These components coat starch granules, hindering their capacity to swell. Moreover, proteins and soluble fiber can also compete with starch granules for water. These effects impede proper gelatinization, thus decreasing their digestion (Diez‐Sánchez et al. 2018; Ye et al. 2018). Additionally, lipids may also form amylose‐lipid complexes in the rice flour that could, to some extent, inhibit the ability of the digestive enzymes to hydrolyze starch (Ye et al. 2018).

The changes in starch digestibility profiles determined the pGI of the samples. All enriched samples presented significantly lower pGI in comparison to the control. The pGI values were 85.1 ± 0.6 for the control, 82.4 ± 0.7 for T10, 78.9 ± 0.9 for T15, 82.0 ± 0.2 for L10, and 78.1 ± 0.4 for L15, displaying a good relationship with the by‐products content: a higher level of tomato or linseed waste were associated with lower pGI values. The in vitro determination of bioavailable carbohydrates in food products has important implications for consumers (Singh et al. 2010). This is especially crucial for individuals following special dietary regimes where low glycemic index foods are used to manage some chronic diseases (e.g., type 2 diabetes mellitus, metabolic syndrome, obesity). Thus, developing GF products with lower pGI and available carbohydrates but also higher protein and fiber content contributes to increasing the variety of healthier food options for people with celiac disease.

## 4 Conclusion

This study successfully demonstrates the potential of utilizing food industry by‐products, such as tomato waste and linseed cake, to enhance the nutritional value of gluten‐free pasta. The study evaluated aspects of the enriched cooked pasta that have not been fully explored in prior studies, such as the cooking and textural parameters, starch digestibility, and total dietary fiber. In this regard, the results show that despite the nutritional improvements, the use of these by‐products affects certain quality parameters, with the impact varying based on the type and concentration of the by‐product.

Specifically, adding linseed cake powder more effectively improves the resistant starch content and better maintains the cooking quality by limiting the cooking loss than the tomato waste powder. Conversely, tomato waste powder‐enriched pasta had a higher total dietary fiber content, allowing the pasta to be marketed as “high fiber” according to EC regulations. Furthermore, all enriched pasta samples, regardless of the by‐product or concentration, showed a significant reduction in their predicted glycemic index (pGI). These findings are particularly relevant for developing healthier products for individuals with celiac disease or those seeking a low‐glycemic‐index diet to manage chronic conditions like diabetes or obesity. The decrease in pGI was achieved through a reduction in rapidly digestible starch (RDS) and an increase in resistant starch (RS).

In summary, this research not only validates the use of food industry by‐products for the functionalization of gluten‐free pasta but also provides insights into how these by‐products impact the quality parameters of cooked pasta and its nutritional value, thereby supporting circular economy schemes in food production.

## Author Contributions

**Gabriella Pasini:** conceptualization (equal), funding acquisition (equal), resources (equal), supervision (equal), validation (equal), visualization (equal), writing – original draft (equal), writing – review and editing (equal). **Carlos Gabriel Arp:** formal analysis (equal), visualization (equal), writing – original draft (equal), writing – review and editing (equal). **Sabrina de Leo:** data curation (equal), investigation (equal), methodology (equal). **Andrea Brandolini:** formal analysis (equal), writing – original draft (equal), writing – review and editing (equal). **Lorenzo Estivi:** data curation (equal), formal analysis (equal), investigation (equal), methodology (equal). **Alyssa Hidalgo:** conceptualization (equal), data curation (equal), formal analysis (equal), resources (equal), validation (equal), writing – original draft (equal), writing – review and editing (equal).

## Conflicts of Interest

The authors declare no conflicts of interest.

## Data Availability

Data available on request from the authors.
